# Antifungal potential of lipopeptides produced by the *Bacillus siamensis* Sh420 strain against *Fusarium graminearum*

**DOI:** 10.1128/spectrum.04008-23

**Published:** 2024-03-07

**Authors:** Sarfaraz Hussain, Bowen Tai, Maratab Ali, Israt Jahan, Suha Sakina, Gang Wang, Xinlong Zhang, Yixuan Yin, Fuguo Xing

**Affiliations:** 1 Key Laboratory of Agro-products Quality and Safety Control in Storage and Transport Process, Ministry of Agriculture and Rural Affairs/Institute of Food Science and Technology, Chinese Academy of Agricultural Sciences, Beijing, China; 2 College of Agricultural Engineering and Food Science, Shandong University of Technology, Zibo, Shandong, China; 3 Department of Agriculture and Food Technology, Karakoram International University, Gilgit-Baltistan, Pakistan; 4 Shandong Xinfurui Agriculture Science Co., Ltd, Liaocheng, Shandong, China; University of Debrecen, Debrecen, Hungary

**Keywords:** *Bacillus siamensis*, lipopeptides, antifungal activity, *Fusarium graminearum*, ergosterol, antioxidant activity

## Abstract

Biological control is a more sustainable and environmentally friendly alternative to chemical fungicides for controlling *Fusarium* spp. infestations. In this work, *Bacillus siamensis* Sh420 isolated from wheat rhizosphere showed a high antifungal activity against *Fusarium graminearum* as a secure substitute for fungicides. Sh420 was identified as *B. siamensis* using phenotypic evaluation and 16S rDNA gene sequence analysis. An *in vitro* antagonistic study showed that Sh420’s lipopeptide (LP) extract exhibited strong antifungal properties and effectively combated *F. graminearum*. Meanwhile, lipopeptides have the ability to decrease ergosterol content, which has an impact on the overall structure and stability of the plasma membrane. The PCR-based screening revealed the presence of antifungal LP biosynthetic genes in this strain’s genomic DNA. In the crude LP extract of Sh420, we were able to discover several LPs such as bacillomycin, iturins, fengycin, and surfactins using ultra-high-performance liquid chromatography–quadrupole time-of-flight mass spectrometry. Microscopic investigations (fluorescent/transmission electron microscopy) revealed deformities and alterations in the morphology of the phytopathogen upon interaction with LPs. Sh420 LPs have been shown in grape tests to be effective against *F. graminearum* infection and to stimulate antioxidant activity in fruits by avoiding rust and gray lesions. The overall findings of this study highlight the potential of Sh420 lipopeptides as an effective biological control agent against *F. graminearum* infestations.

**IMPORTANCE:**

This study addresses the potential of lipopeptide (LP) extracts obtained from the strain identified as *Bacillus siamensis* Sh420. This Sh420 isolate acts as a crucial player in providing a sustainable and environmentally friendly alternative to chemical fungicides for suppressing *Fusarium graminearum* phytopathogen. Moreover, these LPs can reduce ergosterol content in the phytopathogen influencing the overall structure and stability of its plasma membrane. PCR screening provided confirmation regarding the existence of genes responsible for biosynthesizing antifungal LPs in the genomic DNA of Sh420. Several antibiotic lipopeptide compounds were identified from this bacterial crude extract using ultra-high-performance liquid chromatography–quadrupole time-of-flight mass spectrometry. Microscopic investigations revealed deformities and alterations in the morphology of *F. graminearum* upon interaction with LPs. Furthermore, studies on fruit demonstrated the efficacy of Sh420 LPs in mitigating *F. graminearum* infection and stimulating antioxidant activity in fruits, preventing rust and gray lesions.

## INTRODUCTION

Fusarium head blight (FHB) is a major disease caused by *Fusarium graminearum (Fg*) that contributes to severe economic losses to wheat and barley crops globally by reducing cellulose, amylose, and protein, reducing yield and productivity (1). Moreover, its secretory mycotoxin deoxynivalenol leads to feed refusal, gastrointestinal dysfunction, vomiting, and reduced immune functions (2). Controlling this toxin-producing pathogen is a significant challenge regarding food safety and food security. *Fusarium* fungus can infect plants through their roots, seeds, and wounds, especially if the cells that line the root cap are damaged (3).

Although different approaches for controlling FHB are used, removing or burying agricultural wastes infected with *Fusarium* after harvest is problematic due to limited tillage approaches (4). The utilization of host resistance as a means of reducing FHB is a cost-effective and environmentally friendly method, but so far, only a few extremely resistant wheat cultivars have been identified. Foliar fungicides used during anthesis can aid in scab reduction (5). Because fungicides have detrimental impacts on both human health and the environment, the cost of fungicides is rising, and more significantly, some resistant disease-causing species of fungi may be promoted (6). In the same way, crop rotation is also not an efficient technique to reduce FHB infestation (7). Hence, these strategies have some limitations in one way or another.

Recently, the cost-effective, efficient, and environmentally friendly nature of biological control has made this research a hot topic to inhibit the broad spectrum of fungal pathogens (8). Biocontrol techniques can assist growers in lowering their use of chemicals, hence reducing the development of fungicide resistance in pathogen populations (9). Antagonistic bacteria use diverse mechanisms for the biocontrol of *Fusarium*, including siderophore-mediated competition for iron, antibiotic production, and induced systemic resistance (10, 11). Many novel fungal biocontrol bacterial species have been discovered recently, which have antagonistic properties against *F. graminearum* and detoxification activity against its mycotoxin, and researchers are still looking for new strains. Among biocontrol agents, *Bacillus* species (12), lactic acid bacteria (13), and *pseudomonads* (14) are the most studied and well-known antibiotic agents due to their natural ability to create endospores and resistance to harsh environmental conditions. The antibiotic activity could be linked to nutrient competition, hydrolytic enzymes, production of antibiotics, and lipopeptides (LPs). These factors are thought to be the main mode of antifungal action by the antagonistic bacteria (15–17). According to gene sequencing, it has been discovered that over 4% of the genes present in *Bacillus* genome participate in the synthesis of antimicrobial compounds (18). Antimicrobial substances are typically produced through either ribosomal or non-ribosomal synthesis. Ribosomal synthesis gives rise to antimicrobial proteins, bacteriocin, bacteriocin-like inhibitory substances, and subtilin (19), while non-ribosomal peptide synthetases and polyketide synthases are responsible for the production of cyclic lipopeptides belonging to the surfactin, iturin, and fengycin families, as well as polyketides from the difficidin, bacillaene, and microlactin families (20, 21). *Bacillus* species *like Bacillus subtilis, Bacillus pumilus, Bacillus amyloliquefaciens, and Bacillus licheniformis* have been reported to produce LPs with high antifungal activity (22–24). LPs are synthesized by a complex of multienzymes called non-ribosomal peptide synthetases (NRPSs). These low-molecular-weight compounds typically consist of short sequence peptides, with generally less than 50 amino acids. Most of the identified LPs are antimicrobial peptides. NRPSs can generate a diverse range of variants based on their amino acid sequence and fatty acid chain length. In *Bacillus* species, multiple families of LPs and their isoforms can be produced simultaneously.

In this study, we identified *Bacillus siamensis* Sh420 (GenBank accession number SUB13288549), a gram-positive, rod-shaped, aerobic, and motile bacterium, as a potential inhibitor of *F. graminearum*. According to recent studies, *B. siamensis* NKIT9 lipopeptide extract inhibited the growth of *Rhizoctonia solani* (25). Two lipopeptides like iturin A and bacillomycin F from *B. siamensis* JFL15 showed good antifungal activity against *Colletotrichum nymphaeae*, *Magnaporthe grisea,* and *Rhizoctonia solani* (26). However, the antifungal potential of LPs produced by *B. siamensis* against *F. graminearum* has not yet been systematically studied.

Thus, the main goals of this study were to (i) isolate and identify bacterial strains with promising antifungal potential against *F. graminearum*, (ii) identify LPs synthesizing genes in the bacterial genome, (iii) identify antifungal LPs in the crude extract of Sh420 on the basis of molecular weight using ultra-high-performance liquid chromatography–quadrupole time-of-flight mass spectrometry (UPLC-QTOF-MS), (iv) determine fungicidal activity of LPs to disrupt cell membrane structures and its effect on ergosterol content of *Fusarium*, and (v) examine alterations in the morphology of *Fusarium* upon interaction with these LPs using a fluorescent microscope. Additionally, the grape fruit was used to assess the biocontrol capability of LPs, demonstrating how well they could protect the fruit against fungus while also measuring the antioxidant activity and total phenolic content of the lipopeptide-treated fruit. To the best of our knowledge, this is the first report to report the effectiveness of LP antifungal activity against *F. graminearum*, improvement of grape shelf life after LP treatment, and alterations in antioxidant activity and total phenolics in fruits because of LPs.

## MATERIALS AND METHODS

### Plant pathogenic fungi

*Fusarium graminearum* PH1 was obtained from the Institute of Plant Protection, Chinese Academy of Agriculture Science, Beijing, China. This pathogen was preserved in 30% glycerol at −80°C and available for future experimental use.

### Isolation, selection, and characterization of bacterial strain

Bacterial strain was isolated from 35 root soil samples taken from the wheat-producing Pinggu and Langfang regions of China. Isolation was carried out with slight modifications in a previous method described by reference (27). A volume of 10 µL of each bacterial dilution solution was spread on Liquid Broth (LB) plates and incubated at 30°C for 1 day. The plates were then sprayed with spores of *F. graminearum* and incubated at 28°C for 5 days. Bacteria that showed inhibition of fungus were identified as antagonistic against *F. graminearum*. The serially diluted culture was then streaked onto nutrient agar (NA) plates and incubated at 28°C until single colonies appeared. Single colonies were picked and purified by streaking on fresh NA plates. A collection of about 290 strains was secured, and LPs were extracted from that strain that had high antifungal activity after screening. Pure bacterial isolates were grown in LB broth and kept at −80°C with the addition of 30% (wt/vol) glycerol for further use.

For the identification of the bacterial strain, genomic DNA was extracted, and PCR reaction of the 16S rDNA gene fragment was done with the universal primers. The obtained sequences were compared with existing sequences in GenBank by using the BLASTN tool and the phylogenetic tree constructed using similar sequences that were taken from NCBI with MEGA 7 based on the neighbor-joining method, and to evaluate the reliability of the tree, 1,000 bootstrap replications were performed accordingly (28). The inhibitory ratio was calculated by using the following formula:


$$Inhibition\;(\%)=\frac{mycelial\;growth\;(starin\;treated)}{mycelial\;growth\;(no\;stran)}\times 100\%$$

### Morphological and physiological characteristics of Sh420 bacterial isolate

A 15-µL culture was streaked onto a LB agar plate and then incubated at 37°C for 1 day. The resulting colony patterns were then examined using a stereomicroscope. For scanning electron microscopic (SEM) examination, bacterial culture broth grown for 1 day is obtained, centrifuged to collect specimens as a pallet, and then washed twice with phosphate buffer. It was then fixed in a 3% glutaraldehyde buffer and stored at 4°C for 1 day. In order to evaluate the colony and cell morphology of Sh420, we followed reference (29).

### Culture conditions for maximum lipopeptide production and extraction of antifungal lipopeptides from Sh420

*B. siamensis* cells were incubated for 72 h at 37°C by shaking at 180 rpm in a specific medium optimal for lipopeptide production (MOLP), as previously used by reference (30), and MOLP media continued to be the preferred medium for subsequent studies.

LPs were isolated by the acid precipitation method with some modifications as described by reference (31). The strain was incubated in 1,000 mL of MOLP medium at 37°C at 180 rpm for 72 h. Then, it was centrifuged at 4°C at 10,000 rpm for 15 min. The supernatant was collected, and the pH was adjusted to 2 by adding 6 mol/L HCl, which appeared as a white precipitation. Now, this supernatant was put at 4°C after 24 h. It was centrifuged, the supernatant obtained was discarded, and the pellet was kept. Ten to fifteen milliliters of methanol solution was added to it, and again, pH was adjusted to 7.0 with 6 mol/L NaOH. The solution was dried with a rotary vacuum evaporator, and the resulting pellet was filtered using 0.22-µm sterile filter to obtain the lipopeptide crude extract.

### Antifungal activity of the lipopeptides produced by Sh420

Different concentrations of lipopeptide extract solution, such as 0.5, 2, 4, 5, 7, 8, and 10 mg mL^−1^ (wt/vol), in distilled water were prepared to test the antifungal activity. A 2-day fungal mycelial plug was placed in the center of 90-mm plates, LPs were inoculated in wells to one side, and distilled water was loaded into the control well on the edges. The plate is then incubated at 28°C. After 7 days, mycelial growth inhibition was determined by the following formula:


$$Mycelial\;Inhibition\;(\%)=\frac{Diameter\;of\;mycelia\;(LPs\;treated)}{Diameter\;of\;control\;mycelia}\times 100\%$$

### Determination of minimal inhibitory concentration and minimal fungicidal concentration of the lipopeptides

Minimal inhibitory concentration (MIC) is the lowest concentration of lipopeptides required to inhibit fungal growth, while minimal fungicidal concentration (MFC) is the smallest concentration of lipopeptides capable of killing the fungi under standardized laboratory conditions. The experiment was carried out in liquid medium according to the protocol described in section “Culture conditions for maximum lipopeptide production and extraction of antifungal lipopeptides from Sh420”. A volume of 500 µL *F*. *graminearum* spore suspension of concentration 7 × 10^6^ conidia mL^−1^ was poured in 1,200-µL Potato Dextrose Broth (PDB) media. Different concentrations of lipopeptides are used against the fungus growth in a 24-well plate to check MIC after 6 days. The plates were incubated at 28*°*C for 7 days. The results were obtained by observing the presence or absence of fungal growth. The whole content from each well, where there was no growth of *F. graminearum,* was passed to tubes with PDB medium and incubated at 25*°*C for 7 days for the determination of MFC. The protocol was designed with some modifications to reference (32).

### Determination of ergosterol by UV spectrophotometry

Ergosterol content in the cell membrane of *Fg* is investigated by a method as reported previously by reference (33). A volume of 100 µL of 10^7^ spore/mL of *F. graminearum* spore suspension was added to PDB media containing 0, 1, 2, and 4 mg/mL of lipopeptide extract. The mixture was then incubated for 5 days at 28°C. After the incubation period, the cells were collected using centrifugation and suspended in an alcoholic KOH solution (25% wt/vol, 3 mL) for 1 h at 85°C in a water bath. Once the samples were cooled to ambient temperature, sterols were isolated by adding 1 mL of distilled water and 3 mL of n-heptane. The mixture was then vortexed for 3 min to separate the heptane layer, which was subsequently diluted with ethanol before undergoing a spectrophotometric analysis at 240–300 nm wavelengths.

### PCR assay for screening of lipopeptide genes in bacterial isolate

Sh420 was screened for the presence of genes encoding various LPs. The PCR parameters were adjusted as done by reference (34). Each reaction was done in triplicate; a negative control was also included in the PCR amplification. The amplified PCR product from each reaction was visualized on 1% agarose gel. Lipopeptide-specific primers and their fragment size used in this study to amplify LP’s biosynthetic genes are enlisted in Table S1.

### UPLC-QTOF-MS analysis of antifungal lipopeptides

The lipopeptide crude extract used for inhibition is utilized to identify compounds. One-milligram crude extract was dissolved in 2 mL of 10% methanol and analyzed by UPLC (Acquity UPLC R BEH300, Waters) coupled with high-definition mass spectrometry (Waters synapt G2-si UPLC-QTOF/MS). Mass spectrometry was carried out by positive ionization electrospray. The obtained data were processed by the MassLynx software (Waters).

### Microscopic visualization of mycelia treated with lipopeptides using a fluorescent microscope and transmission electron microscopy

A bright field, fluorescent microscope (Himedia) was used to observe *Fusarium* hyphal and spore structures after treatment with LP extract. Fluorescent stains such as propidium iodide (PI) (stains only dead cells) and calcofluor white (CFW) (Sigma-Aldrich) were used to study the fungal mycelia and spores under fluorescent microscopy (Nikon, Japan). A 30-µmol concentration of PI and CFW was prepared separately from their respective stock solutions. A combination of PI and CFW was prepared in a 1:1 ratio, and 20 mL from both stains was used separately for fungal staining. After putting the combination of stains on mycelial pieces on a slide, treated and control samples were incubated for 6 min in a dark room at 25°C before being examined under a fluorescent microscope. All selected fungal tissues were observed using fluorescent microscopy (Nikon DAPI-FITC-TRITC filter combinations).

For transmission electron microscopy (TEM) investigation, hyphae were fixed for 4 h at room temperature in 2% glutaraldehyde, washed four times with 0.1 M phosphate buffer, and then fixed for 2 h with 1% osmium tetraoxide. The hyphae were coated with gold and palladium using a Nanotech sputter coating device and were then examined using a SEM (HITACHI, S-570, Japan) operating at 15 kV as previously stated by Zhao et al. (35).

### Biocontrol assay against *F. graminearum* in fruits

Conidia were harvested from mature *Fusarium*. The surface of the grape fruit was sterilized with 5% NaOCl for 5 min and rinsed three times with plenty of sterile water. Wounds of 3 mm were made with a sterile scalpel on the surface of the grapes; then, 12 mL of a 10 mg mL^−1^ solution of lipopeptides was applied to the injuries and sprayed on the surface of the treated samples. One hour later, when the fruit was dried at room temperature, 0.5 mL of a suspension of *F. graminearum* 10^7^ conidia mL^−1^ was inoculated into the wounds. Fruits treated with sterile water act as negative control. Fruits treated with *F. graminearum* act as positive control. All the treatments were incubated at 25°C, 70% humidity for 5 days. For each treatment, a total of nine fruits were used, and three technical replicates were performed. Results were calculated by the method described in Table 1. The effect was measured and expressed as disease incidence (% of infected fruit).

**TABLE 1:** Fungal severity calculation scale on grape berries

Grade	Symptoms
0	No infection
1	Very small spot
2	One infected spot
3	Two or four infected spots
4	<50% berry was infected, and sporulation was evident
5	>50% berry was infected, and sporulation was evident

### Antioxidant activity and estimation of total phenolics of fruit treated with lipopeptides

A weight of 0.5 g of lyophilized fruit sample was mixed with 6 mL of 80% methanol and 0.1% hydrochloric acid, vortexed to obtain a homogeneous mixture, and then stored for 4–5 h at 4°C to assess the fruit’s antioxidant potential and phenolic activity. The supernatant was filtered, and the extract was used for the determination of the phenol content and the Ferric Reducing Antioxidant Power Assay (FRAP). In a test tube, 1 mL of the sample was mixed with 2 mL of phosphate buffer and 2.5 mL of potassium ferricyanide solution, and then, the test tube was wrapped in an aluminum foil. Afterward, the test tube was incubated in a water bath at 50°C for 20 min. After shaking, 2.5 mL of 10% trichloroacetic acid was added and centrifuged at 3,000 rpm for 5–6 min. The upper 2.5 mL of supernatant was taken to a new test tube. One milliliter of distilled water was added to the new tube. Now, 0.5-mL ferric chloride was added to that upper layer, and a thorough mix was given. A bluish compound formation was obtained. Now, 200 µL was taken from the tubes and poured into a 96-well plate. Absorbance optical density (OD) was taken at 700 nm. The higher the absorbance, the higher the antioxidant activity. Ascorbic acid was used as positive control. The percentage of antioxidant activity was calculated using the formula:


$$Antioxidant\;activity\;(\%)=(A_{control}-A_{sample})÷A_{control}\times 100\%$$

For total phenolic estimation, 100 µL of the sample extract and 400 µL of distilled water were added and mixed to dilute the extract, and 150 µL of FC reagent (diluted with distilled water in the ratio 1:1 vol/vol) was added. After vortexing, it was kept at room temperature for 5 min. Then, a bluish color compound was formed by adding 500 µL sodium carbonate and incubating in the dark for 1 h. Then, a microplate reader was used to read the absorbance at 650 nm (Bio-Rad, iMARK, Japan). For 500 µL of methanol, all the reagents were added except fruit extract and considered as blank. Gallic acid was taken as a standard to determine the phenolic content of the samples.

### Statistical analyses

CRD design was implemented to conduct the experiment. The data collected were analyzed using ANOVA, for comparing means between groups. Following this, Duncan’s multiple range test was used to make comparisons between pairs of groups.

## RESULTS

### Antagonistic activity and identification of bacterial isolate

**Fig 1:**
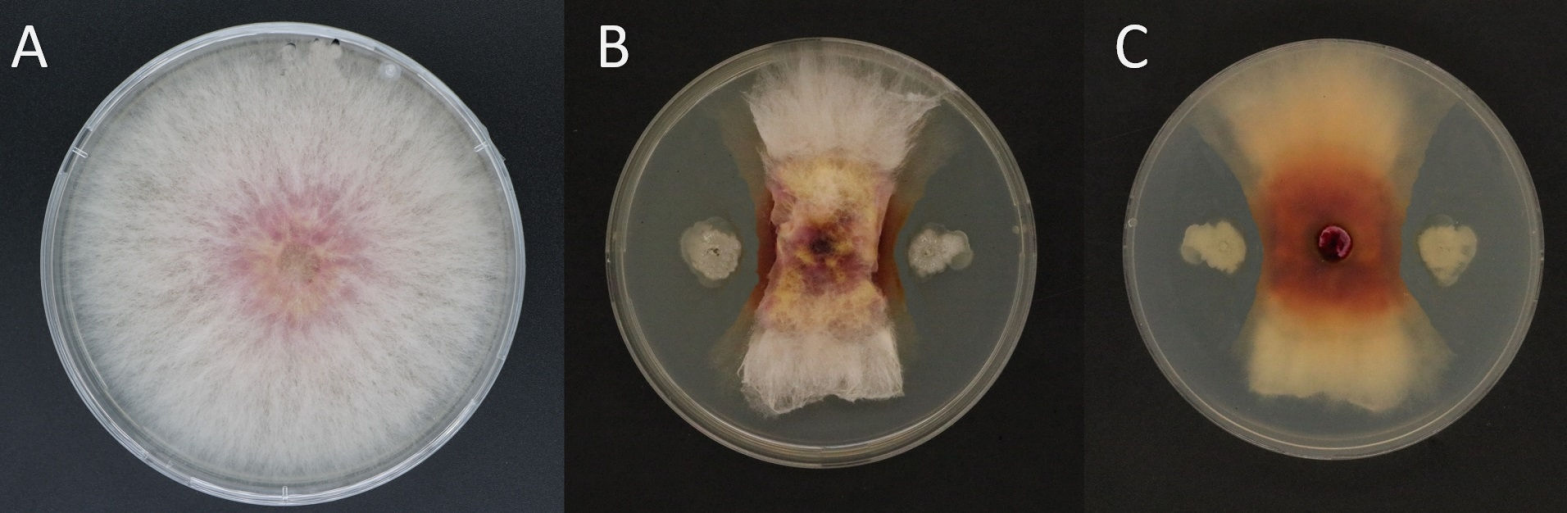
Antifungal activity of *B. siamensis* Sh420 against *F. graminearum* in dual culture plate on PDA media at sixth of incubation at 28°C. (**A**) CK, a 5-mm agar plug of *F. graminearum* at the center of PDA plate and (**B**) front side of plate where Sh420 is inoculated on two sites that are 2.5 cm apart from the *F. graminearum* colony. (**C**) Back side of the dual culture plate.

In this research, over 290 bacterial strains were isolated from soil samples of wheat rhizosphere. After screening, we selected Sh420 strain for further analysis, as it exhibited significant antagonistic activity against *F. graminearum* in a dual culture method and observed that 5 µL of Sh420 with 10^7^ CFU/mL effectively inhibited the mycelia growth indicated by the inhibition zones on Potato Dextrose Agar (PDA) (Fig. 1).

After analyzing the 16S rDNA gene sequence of the isolates using blast software (NCBI database), it was determined that the isolate Sh420 belongs to the *Bacillus* genus having a correlation of 99.78% with known species (Fig. S1). Hence, it is identified as *B. siamensis*, which is a gram-positive, rod-shaped bacterium. It can produce a range of enzymes and bioactive compounds. Further study is required to truly understand its possible applications. In a previous study, the LZ88 strain of *B. siamensis* showed an 81.96% inhibition rate, against brown spot disease in tobacco, caused by *Alternaria alternata* (36). The partial 16S rDNA gene sequences of strain Sh420 were added to the GenBank database with accession no. SUB13288549.

### Morphological and physiological characteristics of *Bacillus siamensis* Sh420

**Fig 2:**
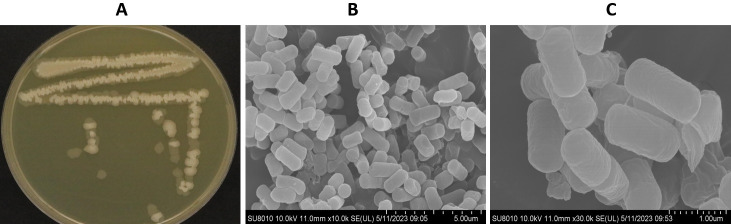
Morphological features of *B. siamensis* Sh420. (**A**) Colony morphology. (**B**) Bacterial cells in chain form. (**C**) Single bacterium cells.

The *B. siamensis* Sh420 colony had a slightly rough texture and was off-white to creamy in color with well-defined and clear colony edges on LB agar. The colonies were linked together and appeared as a chain when viewed via a stereomicroscope. SEM revealed that Sh420 cells were straight and rod-shaped with round ends, organized in chains, and motile (Fig. 2). The bacterial cell was 1–1.7 µm in length and 2–5 µm in width.

### Antifungal activity of lipopeptide and screening of lipopeptide genes

**Fig 3:**
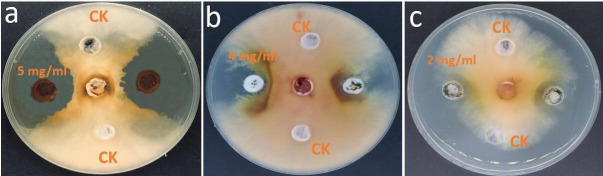
Antifungal activity of *B. siamensis* Sh420 lipopeptides against *F. graminearum*. Assessment of different concentrations of LPs against fungal growth: reverse side of Petri plates showing inhibition zones and control in the same plate: (**A**) 5 mg mL^−1^ LPs, (**B**) 4 mg mL^−1^ LPs, and (**C**) 2 mg mL^−1^ LPs.

LPs showed antifungal activity against *F. graminearum* in the well inoculation method. Inhibition rates of 70%, 62%, and 40% of the mycelia diameter were observed for the concentrations of LPs of 5, 4, and 2 mg mL^−1^, respectively, after 7 days of treatment (Fig. 3). In general, LPs from Sh420 showed antagonistic activity against *F. graminearum* across a broad spectrum of concentrations in a dose-dependent manner. LPs were screened using certain primers that have been previously researched in *Bacillus* species-related literature. From these specific genes, we identified many important lipopeptide genes using PCR reaction; those include Loap (antiterminator protein that regulates antibiotic gene clusters), FenB (fengycin), ituA (iturin), itu D (iturin D), BmyA (bacillomycin A), dhbA (bacillibactin), sfp (surfactin), srfA (surfactin), dfnA (difficidin), dfnM (difficidin), bacA (bacilysin), and beaB (bacillaene). These antibiotic genes were detected on gel visualization (Fig. S2).

### Determination of MIC and MFC of the lipopeptides

**Fig 4:**
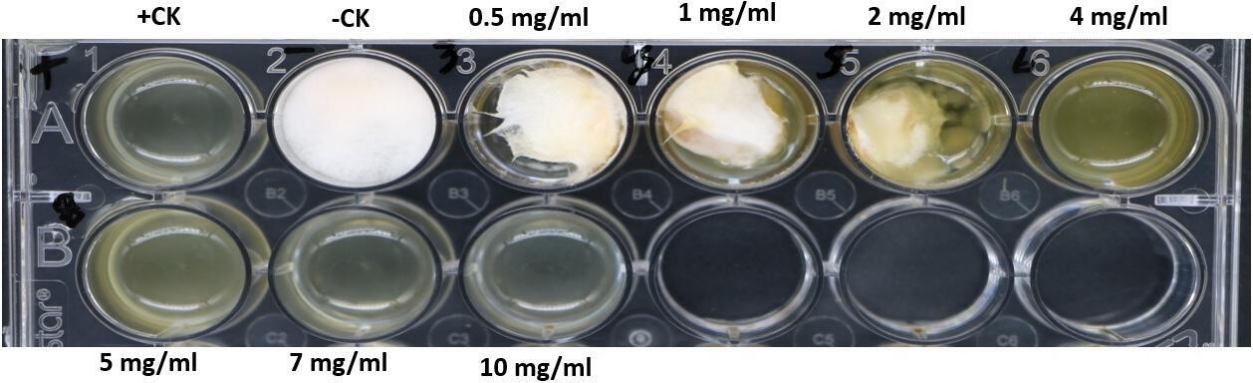
MIC and MFC of lipopeptides from Sh420. +CK (positive control) medium inoculated with amphotericin B for fungal growth inhibition, while −CK (negative control) PDB inoculated with spore suspension without any treatment.

Lipopeptides from Sh420 were tested against *F. graminearum* in 24 multiwell culture plates to check the MIC and MFC. Different concentrations of lipopeptides were tested ranging from 0.5 to 10 mg mL^−1^. In PDB broth, *Fusarium* growth was observed to be decreased and stopped with increasing lipopeptide concentrations. At a lipopeptide concentration of 5 mg mL^−1^, no fungus germinated in PDB after 7 days of incubation. Thus, the MIC of lipopeptide against *Fg* was determined to be 5 mg mL^−1^ (Fig. 4).

### Ergosterol content determination

**Fig 5:**
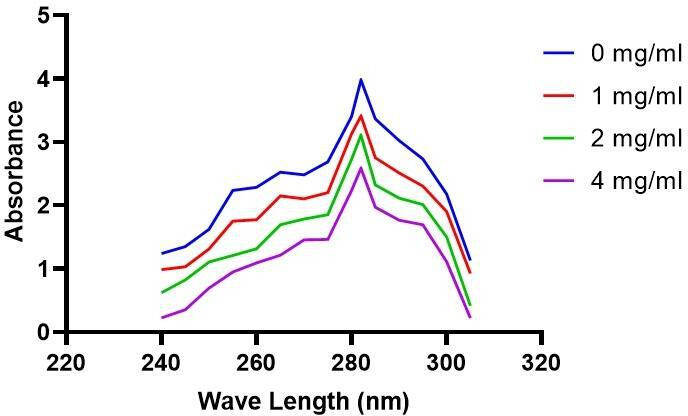
Spectrometric analysis of ergosterol profile of *F. graminearum* at various concentrations of lipopeptides.

Ergosterol is a critical component of fungal membranes and plays a crucial role in maintaining their structure and function (37). To confirm the impact of the lipopeptide extract, the plasma membrane’s ergosterol content was measured. The effects of lipopeptides on the ergosterol content in the plasma membrane of *F. graminearum* are shown in (Fig. 5). The cells were treated with different concentrations of lipopeptide extract (0, 1, 2, and 4 mg/mL), and the results demonstrated that the levels of ergosterol in the *F. graminearum* membranes decreased significantly in a dose-dependent manner, with the maximum inhibition observed at 4 mg/mL. The highest level of absorbance is observed at a wavelength of 282 nm. This finding indicates that lipopeptides have a considerable impact on inhibiting the biosynthesis of ergosterol in *F. graminearum*, disrupting the production of this essential fungal membrane component. This disruption may lead to impaired growth and function of the fungus.

### Identification of lipopeptides using UPLC-QTOF/MS analysis

**Fig 6:**
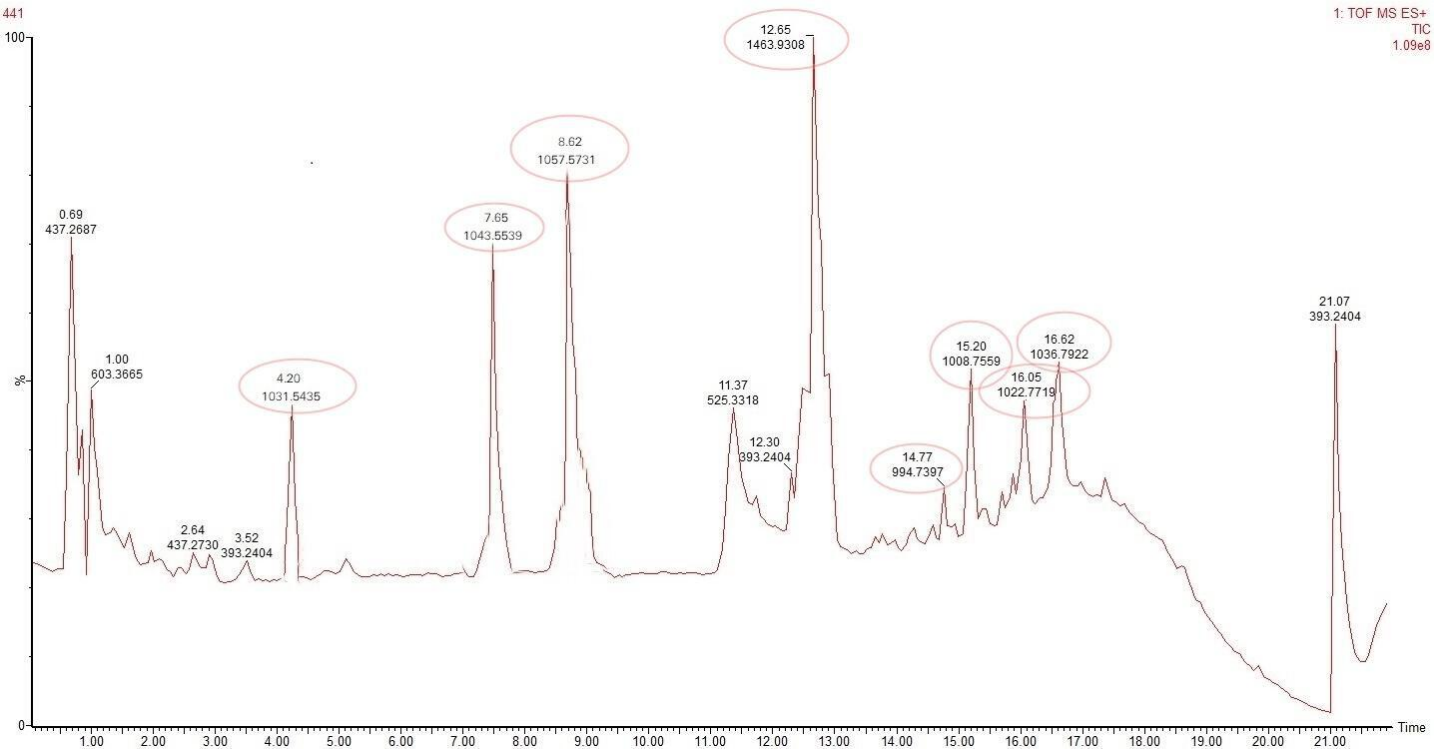
TOF-MS ES + TIC of Sh420 LPs with their molecular weight (m/z) and Rt.

UPLC-QTOF mass spectrometry examination of the purified extract led to the identification of different lipopeptides from various groups. By comparing the mass information obtained from individual peak fractions with the mass data reported for the cyclic lipopeptides from various *Bacillus* species strains, it is possible to identify the lipopeptide from the crude extract of Sh420 using the total ion chromatogram (TIC) spectrum and corresponding (m/z) values of peaks (Fig. 6). The main lipopeptide compounds of strain Sh420 were identified as bacillomycin D (C14) and two known iturins A2 (C14) and iturin A3-A5 (C-15). Fengycin A (C16) is also found in the extract. Four known surfactins with an acyl chain ranging from C12 to C15 were also detected. Analysis using quadrupole time-of-flight mass spectrometry revealed a total of eight main LP compounds from four different classes of lipopeptides. This investigation detected a [M+H] peak at m/z 1,031.5435 with the molecular formula C_48_H_75_N1_0_O_15_ that represents bacillomycin D. Another [M+H] peak at m/z 1,043.5539 afforded the molecular formula C_48_H_74_N_12_O_14_, which represents iturin A, and another [M+H] peak at m/z 1,057.5731 corresponding to iturin A3 with the molecular formula C_49_H_76_N_12_O_14_ is detected. Such similar LP with almost similar mass is reported in previous literature too. At retention time (Rt) of 12.65, a [M+H] peak at m/z 1,463.9308 with the molecular formula C_72_H_110_N_12_O_20_ (i-Fit D 130.3 and DBE D 23.5) conforms to fengycin A. Furthermore, four other surfactin isomers were identified from the extract. At 14.77 retention time, a peak with protonated molecular ion [M+H] at m/z 994.7397 is identified as surfactin with a C12 fatty acid chain. Other surfactins at Rt 15.20, 16.05, and 16.62 with [M+H] at m/z = 1,008.7559, m/z = 1,036, and m/z = 1,022.7719 with the molecular formula C_52_H_91_N_7_O_13_ were identified, respectively (Fig. 6), as reported in previous literature compounds with almost similar mass identified during LP’s studies (Table 2) (38, 39).

**TABLE 2:** Lipopeptide production by *B. siamensis* Sh420 detected by UPLC-QTOF

Lipopeptide	Chain length	Retention time	Current experimental mass/[M+H]^+^	Previously reported mass	Reference
Bacillomycin D	C14	4.20	1,031.5435	1,031.5431	(39)
Iturin C	C14	7.65	1,043.5539	1,043.528	(40)
Iturin A3	C15	8.62	1,057.5731	1,057.5830	(41)
Fengycin A	C16	12.65	1,463.9308	1,463.8185	(41)
Surfactin	C12	14.77	994.7397	994.64	(42)
Surfactin	C13	15.20	1,008.7559	1,008.75	(43)
Surfactin	C14	16.05	1,022.7719	1,022.6729	(39)
Surfactin	C15	16.62	1,036.7922	1,036.6954	(41)

### Effect of Sh420 bacterial strain and its lipopeptides on *F. graminearum* mycelia and spore structures

**Fig 7:**
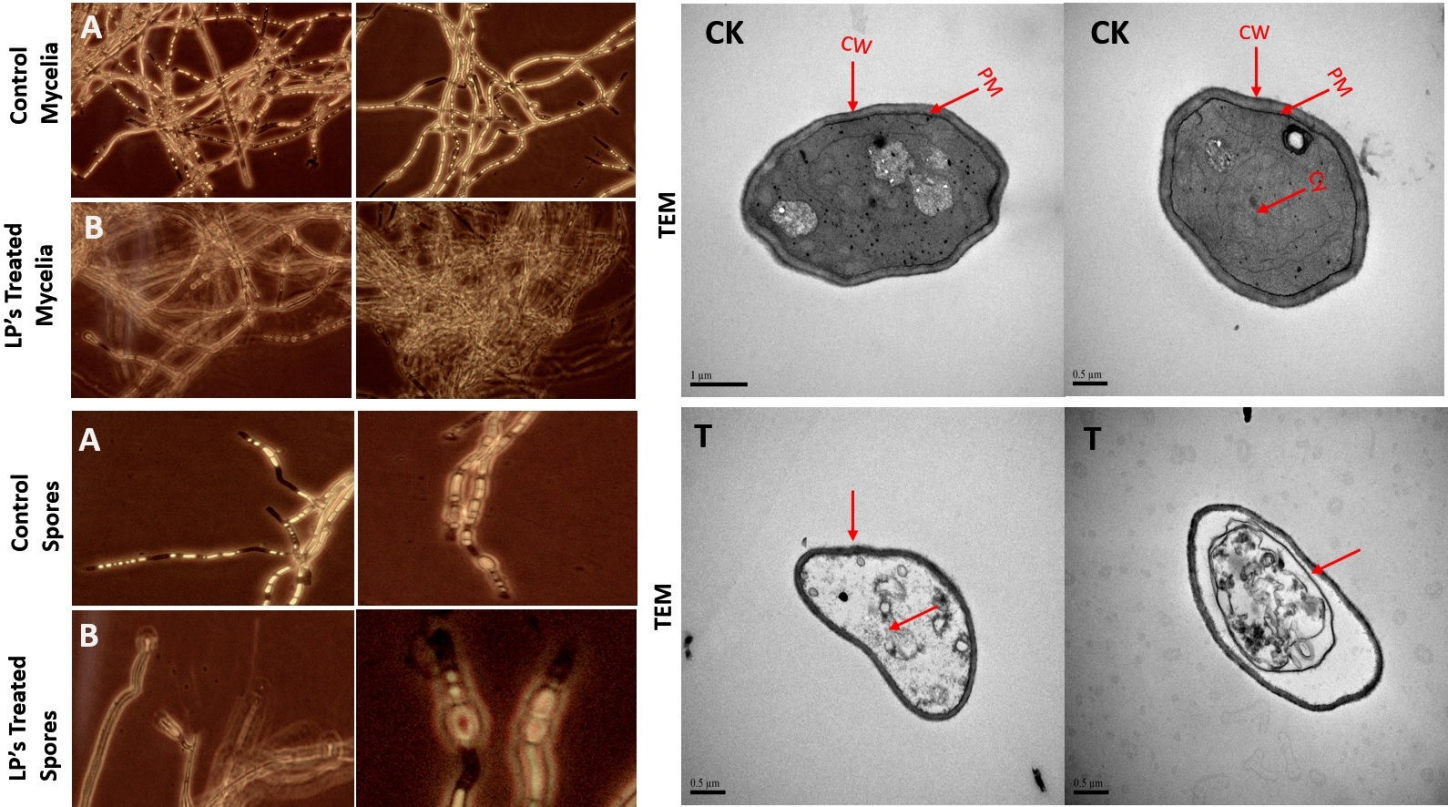
Effects of lipopeptides on *F. graminearum*. Control mycelia and spores (**A**). Treated mycelia and spores (**B**). Treated fungi showed deformation, swelling, and bulging in hyphae and spores, which are clearly visible in bright field burst or swelled portions of the hyphae, or spores had blurred images as compared to controls. TEM photographs of treated *F. graminearum* where red arrows showed the abnormal membranes and cell structure as compared to untreated mycelia.

Lipopeptide extract showed significant inhibitory effect and damaging effects against *F. graminearum* pathogen. Lipopeptide extract effects on *Fusarium* were analyzed using CFW plus PI staining. The chitin contained in the cell walls of fungi is stained by CFW, which distinguishes between live and dead cells, whereas PI exclusively stains dead cells. Luminous dye was used in fluorescent microscopy (Nikon, Japan), which revealed abnormal hyphae and deformations in the fungus. In samples treated with lipopeptide, considerable numbers of dead *Fusarium* hyphae and their spores were seen using CFW staining, whereas no or very few damaged or dead cells were evident in control samples. Spores showed swelling and bulging in the treated samples. Furthermore, disintegration and lysis of spore/cells were also observed. However, smooth and normal structures of spores without any deformation were observed in untreated *Fusarium* (Fig. 7). Burst or swelled portions of the hyphae or spores had blurred images as compared to controls. This may be due to the loss of chitin and glucans from the cell walls. Lipopeptides create pores in fungal hyphae by depolarizing membranes, inhibiting chitin and glucan synthases, and inducing apoptosis. As CFW stained the chitin contained in the cell wall and spores, live fungal mycelia cells and spores were clearly visible, which have intact cell walls; however, burst/ruptured or damaged cells were not clearly visible (blurred) at spore tips and edges. Further analysis using TEM confirmed that Sh420 LPs caused cellular deformities such as loss of integrity, cell wall and plasma membrane damage, cell shrinkage, cytoplasmic displacement, and degeneration of organelles in the fungal hyphae, while control hyphae maintained good cellular shape, dense cytosol, and integrity.

### Biocontrol assays of Sh420 lipopeptides against *F. graminearum* pathogenicity in grapes

**Fig 8:**
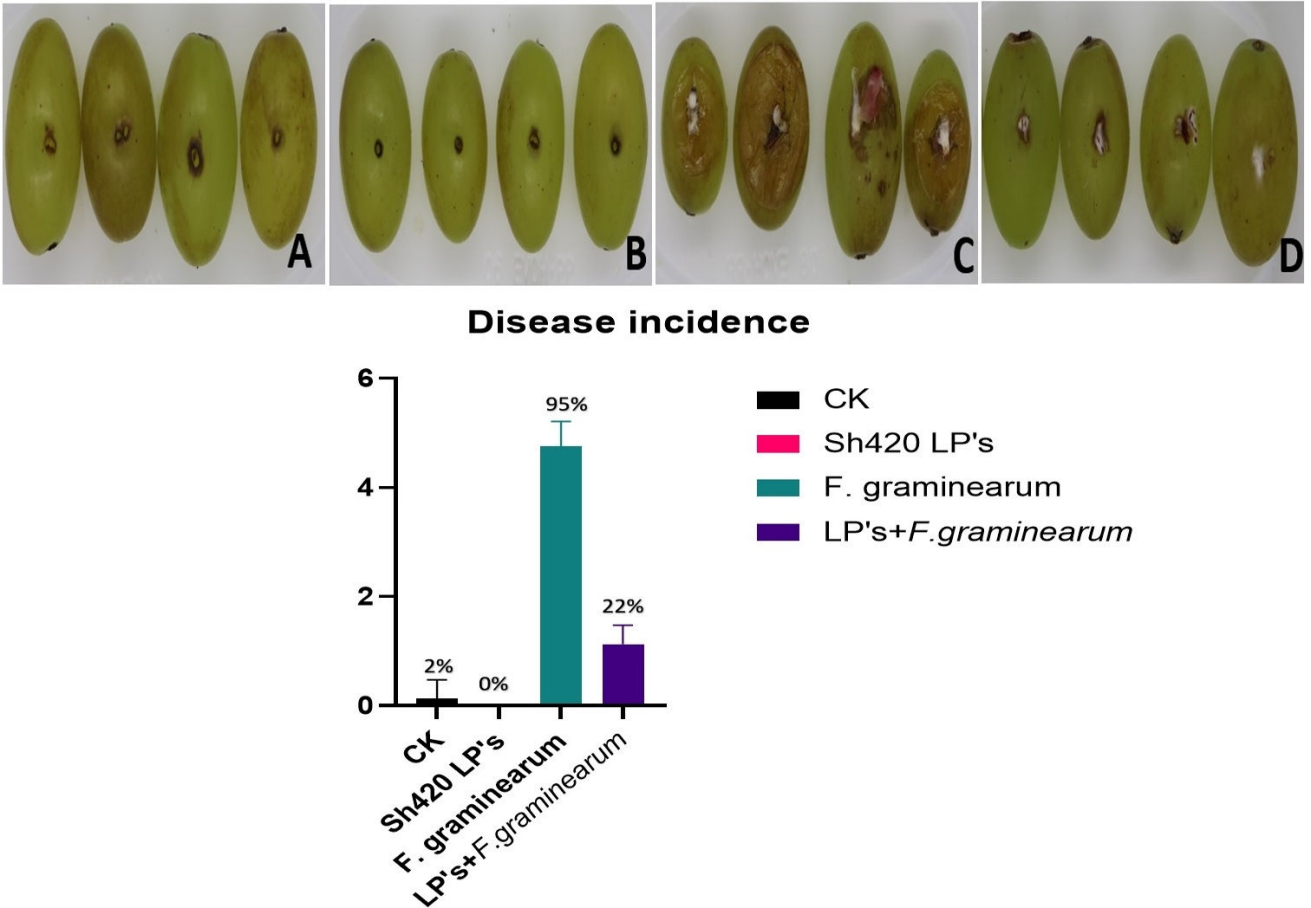
Lipopeptides from Sh420 inhibited disease severity in grapes. Disease severity of gray mold on grapes. (**A**) Grapes treated with sterile water (negative control). (**B**) Grapes treated with LPs. (**C**) Grapes infected with *Fusarium.* (**D**) Grapes infected with both *F. graminearum* and LPs. (**E**) Graphical representation of disease severity in all four groups.

To assess the protective effect of lipopeptides on fruits infected with *Fusarium*, the MIC/MFC of lipopeptide extract from Sh420 (5 mg mL^−1^) was used. The lipopeptides were sprayed on and injected too after surface sterilization. The prevalence of disease infections decreased on fruits treated with Sh420 LPs. The disease incidence in grapes treated with *Fg* was 95%. The disease inhibition in fruits treated with Sh420 LPs was 100%, and no disease symptoms and even gray spots or lesions have appeared on the grapes. On the other hand, the group treated with both LPs and *Fusarium* showed a disease incidence of 22% (Fig. 8). These findings demonstrated that fruits treated with antifungal LP extract have considerably reduced disease symptoms compared to untreated fruits. Overall, the disease incidence declined significantly in grapes.

### Antioxidant activity on fruit treated with lipopeptides

**Fig 9:**
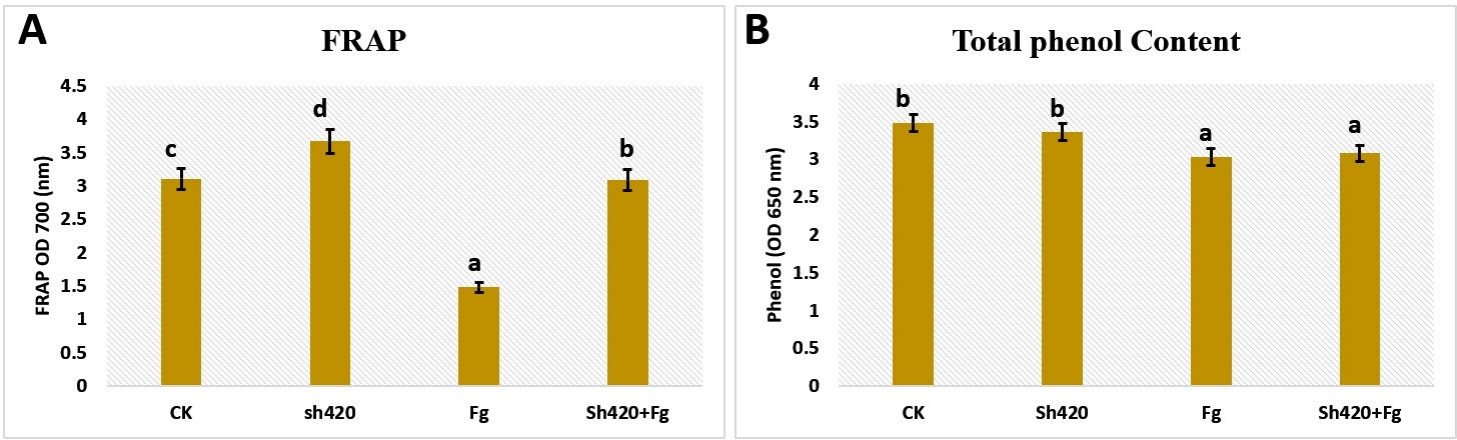
Antioxidant activity evaluated with the FRAP assay (**A**) and total phenols (**B**) of grapes treated with LPs. Control (CK), Sh420 (lipopeptide-treated), *F. graminearum* (*Fg*), *and* LPs*+Fg*. Values followed by the same letters did not differ significantly according to Duncan’s multiple range test (*P <* 0.05). Vertical lines represent the standard errors of the mean.

According to this study, grapes have the highest antioxidant activity. Using MIC of the lipopeptides produced by Sh420 greatly enhanced the antioxidant activity of grape extracts as evaluated by the FRAP experiment (Fig. 9A). The antioxidant activity was highest in LP-treated grapes; however, in the control group, it was relatively low. Antioxidant activity was lowest in grapes exposed to a disease. However, in terms of phenol content, uninfected grapes (CK) showed a significant increase in total phenol content, followed by the LP-treated group. The pathogen-treated group and the grapes treated with both LPs and *Fusarium* pathogen displayed almost the same trend as a result of exposure to lipopeptides (Fig. 9B).

## DISCUSSION

*Bacillus* LPs are a class of secondary metabolites, which possess a wide range of antimicrobial activities, including inhibitory effects against fungi (44). In this study, we isolated and identified the LPs from *B. siamensis* and checked its fungicidal potential against *F. graminearum* that badly affects cereal crops, causing FHB, which leads to significant economic losses worldwide. To avoid these losses and control such pathogens, researchers are looking for various solutions. One of which is the identification of LPs and their potential antibiotic action against various fungal strains. Previous studies have shown that the antifungal action of *Bacillus* species is caused by the production of LPs such as surfactin, iturin, bacillomycin, and fengycin. These LPs are produced by NRPSs (45). These compounds consist of a peptide chain and a lipid tail, making them amphiphilic and able to interact with cell membranes. Their antifungal activity has been attributed to various mechanisms, including disruption of cell membrane integrity, inhibition of cell wall synthesis, and induction of oxidative stress (46).

Our LP extract showed inhibitory activity against *F. graminearum in vitro*. The LP extract showed higher inhibitory activity with the increase of their concentration. Likewise, *B. subtilis* B1 LPs combat the *Lasiodiplodia theobromae* fungus (47). *B. siamensis* LPs displayed antifungal activities against multiple fungi like *M. grisea*, *R. solani*, and *Colletotrichum gloeosporioides* (26). Fengycin lipopeptides from *B. subtilis* BS155 induce membrane damage and organelle dysfunction, disrupt mitochondrial membrane potential, cause oxidative stress, and condense chromatin, ultimately leading to the death of *M. grisea* hyphal cells (48).

Moreover, ergosterol production is significantly disrupted in *F. graminearum* due to lipopeptides. LPs work by obstructing the sterol production process, especially when it comes to ergosterol. These fungal sterols consist of two main compounds, ergosterol and 24 (28)-dehydroergosterol. These sterols exhibit their highest level of absorption at a wavelength of 281.5 nm (33). Our results showed maximum absorption at 282 nm. This characteristic absorption pattern provides valuable insights into determining ergosterol levels. This disruption affects both structural integrity and the proper functioning of the membrane. The overall effect of LPs is the prevention of fungal growth since ergosterol also has a hormone-like effect on fungal cells, triggering growth processes. Similarly, according to a study, the *Fusarium oxysporum f.* sp*. Cubense* ergosterol level is reduced due to two cyclic LPs from *Streptomyces* sp. XY006 (49). Likewise, 4-methyl hexanoyl conjugated trimeric battacin lipopeptide displayed antifungal activity by reducing the ergosterol and suppressing the biofilms of *Candida albicans* (50). Amphotericin B, used against *Candida albicans*, has a similar action by interacting with ergosterol and causing membrane breakdown, intracellular content leakage, and cell death of fungi (51).

In our investigation, the biosynthetic genes for LPs from the genome of *B*. *siamensis* were amplified using specific primers for the detection of various forms of lipopeptides using PCR, which confirmed the presence of gene clusters for various NRP’s lipopeptide while some of the genes are not detected in Sh420. Several current studies have stated that the genomes of *Bacillus* species like *B. amyloliquefaciens* (52)*, B. subtilis* (42)*,* and *B. siamensis* contain biosynthetic genes that express antifungal LPs*,* including bacillomycin, surfactin, fengycin, bacillibactin, iturin, subtilosin, and bacilysin (53).

We identified the MIC of the LP extract, which was as low as 5 mg mL^−1^. Thus, the MIC of lipopeptide against *Fg* was determined to be 5 mg mL^−1^ (Fig. 4). These observations confirm the strong antifungal activity of the extract. In previous studies, MIC/MFC of LPs from *Bacillus methylotrophicus* XT1 was 8 mg mL^−1^ against *Botrytis cinerea* (39). LPs from *B. subtilis* SPB1 showed a MIC of 3 mg mL^−1^ against *Fusarium solani* (54). MICs depend on the type of bacteria and the quantity of lipopeptides in the extract. Likewise, the MIC concentration of a single peptide from *B. amyloliquefaciens* against fungal growth was 30 mg L^−1^ (55). The presence of antifungal LPs in the crude extract of Sh420 was further confirmed by UPLC-QTOF/MS analysis. The LPs were detected based on their mass-to-charge ratio (m/z). The main lipopeptide compounds of strain Sh420 were identified as bacillomycin D, two known iturins, and fengycin A, and four known surfactins with an acyl chain ranging from C12 to C15 were also detected (Table 2). After PCR amplification, many different LPs were shown on the gel, but later, some of these compounds were not found using UPLC-QTOF mass spectrometry analysis. It is possible that their concentrations were too low, in which case their peaks might not have shown. Studies have reported the potential of *Bacillus* species for producing several antifungal LPs against different fungal infections, which were detected by MS based on their molecular weight (40). LPs with antifungal properties against *Sclerotinia sclerotiorum* were identified in a study where LC-MS analysis was conducted on the crude mixture of 47 strains of *Bacillus* (56). The effects of lipopeptides on *F. graminearum* fungal hyphae and spores were visualized under fluorescent microscopy. In LPs-treated fungi, hyphae and spores had deformation, swelling, and bulging that were clearly evident in bright field, while swollen or ruptured sections of the hyphae or spores exhibited fuzzy or blurred pictures. LPs can cause the formation of pores in fungal hyphae by inducing depolarization of membranes, suppressing the synthesis of chitin and glucans, and triggering apoptosis, which could be attributed to the absence of chitin and glucans from cell walls. In a recent study, LPs were found to inhibit the growth of *Verticillium dahliae* by inducing cell lysis and hyphal swelling, as well as downregulating genes related to protein catabolism and secondary metabolism signaling pathways and melanin production (57). Mihalache et al. (58) studied the effects of LPs on *F. oxysporum,* which demonstrated that control hyphae were normal/healthy and exhibited typical conidiophore and microconidia. Conversely, the LP-treated hyphae exhibited shrinkage, perforation, and disintegration with small, curly, and narrow branching (58). Fluorescence microscopy along with SEM showed that *Fusarium* mycelia and spores showed shivering, formation of pores in the membrane, leakage of protoplasmic substances from cells that leads to death of hyphae, and spores due to LP treatment (59). Lipopeptides produced by *B. subtilis* YM have the potential to make fungal spores more permeable, preventing their germination (60). Furthermore, lipopeptides from *B. subtilis* BS-99-H lead to the swelling and distortion of the fungal hyphae in *Pestalotiopsis eugeniae* (61).

In this study, we also evaluated the protective effects of LPs from Sh420 against mold infestation and lesions in fruits (grapes). Since no disease symptoms or lesions were seen on fruits treated with Sh420 lipopeptides, the disease was completely inhibited in fruits treated with LPs, and no lesions or even gray spots were seen on the grapes. Disease incidence in grapes treated only with fungal spores was 95%. Likewise, the grapes treated with both LPs and *Fusarium* showed disease incidence of 22% (Fig. 6). According to earlier studies, grapevines are protected from *B. cinerea* infection and given localized protection by lipopeptides generated from *B. subtilis* (62). Similarly, LPs by *B. subtilis* Y17B possess significant biocontrol abilities to combat *A. alternata* fruit rot in cherry fruit (42). LPs produced are recognized by plant cells through specific receptors and activate different signaling pathways, including the MAPK cascade, leading to the activation of defense-related genes. This recognition and activation of plant defense responses are important in plant–microbe interactions, as they contribute to the plant’s ability to defend against pathogens (63).

This study has found that Sh420 LPs have the potential to activate the antioxidant properties in fruit and protect them from getting rusty/brownish. The greatest increase in antioxidant activity was seen in fruits treated with LPs. By exposing the fruit to LPs produced by Sh420, the total phenolic content was significantly increased in control and LP-treated grapes, followed by the combined treatment of LPs and pathogen. The findings suggest that the antibiotic effects of LPs and the accumulation of antioxidant compounds may be linked to the fruit’s ability to resist pathogens. In short, LPs have a significant antibiotic and biocontrol potential against molds; hence, they can be used for plants for the competitive inhibition of phytopathogens.

### Conclusion

The study focused on examining the effectiveness of Sh420 lipopeptides in preventing the growth of *F. graminearum*, a fungus responsible for causing FHB in grains. It was found that the lipopeptides produced by the strain were able to control the growth of *F. graminearum* by inhibiting ergosterol synthesis and stimulating antioxidant activity in fruits. The results showed that Sh420 had a significant inhibitory potential to control fungal pathogens. It also has the potential to protect the fruits against molds and lesions and act as an oxidant, making it a promising alternative to chemical fungicides for reducing damage caused by *Fusarium*.

## SUPPLEMENTAL MATERIAL

SupplementaL material spectrum.04008-23-s0001.docx Gene primers, phylogenetic analysis, and gel documentation.

ASM does not own the copyrights to Supplemental Material that may be linked to, or accessed through, an article. The authors have granted ASM a non-exclusive, world-wide license to publish the Supplemental Material files. Please contact the corresponding author directly for reuse.
