# Supplementary material for: Antifungal potential of lipopeptides produced by the Bacillus siamensis Sh420 strain against Fusarium graminearum
Source: Microbiol Spectr. 2024 Mar 7;12(4):e04008-23. doi: 10.1128/spectrum.04008-23 (PMC10986469; doi:10.1128/spectrum.04008-23)
Supplement: SupplementaL material — Gene primers, phylogenetic analysis, and gel documentation. [file spectrum.04008-23-s0001.docx]

| Table S1. Lipopeptide screening using specific Primers used in this study to amplify the LP’s biosynthetic genes through PCR | | | | | |
| --- | --- | --- | --- | --- | --- |
| No. | Lipopeptides | LP’S Homologs | Fragment Size (bp) | Primers | Reference |
| 1 | Antiterminator protein regulator of antibiotic gene clusters | *Loap* |  | Loap-F  ATGAAATGGTACGCACTTTTTG  Loap-R  CTAGGGTTTGGAGAGCACT | (Goodson, Klupt, Zhang, Straight, & Winkler, 2017) |
| 2 | Bacillomycin D | *bmyA* | 1200 | bmyA-F  AAAGCGGCTCAAGAAGCGAAACCC  bmyA-R  CGATTCAGCTCATCGACCAGGTAGGC | (张学雯, et al., 2019) |
| 3 |  | *bmyB* | 983 | bmyB-F  AATAGAAGAACTGCTGGCGT  bmyB-R  GCCTTCCCGACACGACACT | (张学雯, et al., 2019) |
| 4 |  | *bmyC* | 875 | BmyC-F  GAAGGACACGGCAGAGAGGTC  BmyC-R  CACTGATGACTGTTCCTGCT | (张学雯, et al., 2019) |
| 5 | Fengycin | *fenA* | 1000 | fenA-F  GCTGTCCGTTCTGCTTTTTC  fenA-R  GTCGGTGCATGAAATGTACG | (张学雯, et al., 2019) |
| 6 |  | *fenB* | 1600 | fenB-F  CTATAGTTTGTTGACGGCTC  fenB-R  CAGCACTGGTTCTTGTCGCA | (张学雯, et al., 2019) |
| 7 |  | *fenD* | 1600 | fenD-F  TTTGGCAGCAGGAGAAGTTT  fenD-R  GACAGTGCTGCCTGATGAAA | (张学雯, et al., 2019) |
| 8 | Iturin | *ItuA* | 1047 | ItuA-F  ATGTATACCAGTCAATTCC  ItuA-R  GATCCGAAGCTGACAATAG | (张学雯, et al., 2019) |
| 9 |  | *ItuB* | 449 | ItuB-F  CAACGGTATCGAAGCA  ItuB-R  CGTCTCGGGTATCATTT | (张学雯, et al., 2019) |
| 10 |  | *ItuC* | 575 | ItuC-F  TTCACTTTTGATCTGGCGAT  ItuC-R  CGTCCGGTACATTTTCAC | (张学雯, et al., 2019) |
| 11 |  | *ItuD* | 647 | ItuD-F  GATGCGATCTCCTTGGATGT  ItuD-R  ATCGTCATGTGCTGCTTGAG | (张学雯, et al., 2019) |
| 12 | Macrolactin | *mlnA* |  | mlnA-F  CGGCTGCGGGGGAAAAGATCCG  mlnA-R  CAGCATCAGGGCGTGTATGACCTTC | (Arguelles-Arias, et al., 2009) |
| 13 |  | *mlnI* |  | mlnI-F  GGAAGAAAAACAGTCGAGGCGATGCTG  mlnI-R  GAGAAGCTCCGCCGTCACCAGTG | (Arguelles-Arias, et al., 2009) |
| 14 | Surfactin | *sfp* | 675 | *sfp-F*  ATGAAGATTTACGGAATTTA  *sfp-F*  TTATAAAAGCTCTTCGTACG | (Shahid, et al., 2021) |
| 15 | Surfactin | *srfA-A* | 419–431 | *srf*A-F  CGCGGMTACCGVATYGAGC  *srf*A-R  ATBCCTTTBTWDGAATGTCCGCC | (Toral, Rodríguez, Béjar, & Sampedro, 2018) |
| 16 | Bacillibactin | *dhbA* |  | dhbA-F  CGCCTAAAGTAGCGCCGCCATCAACGC  DhbA-R  CCGCGATGGAGCGGGATTATCCG | (Arguelles-Arias, et al., 2009) |


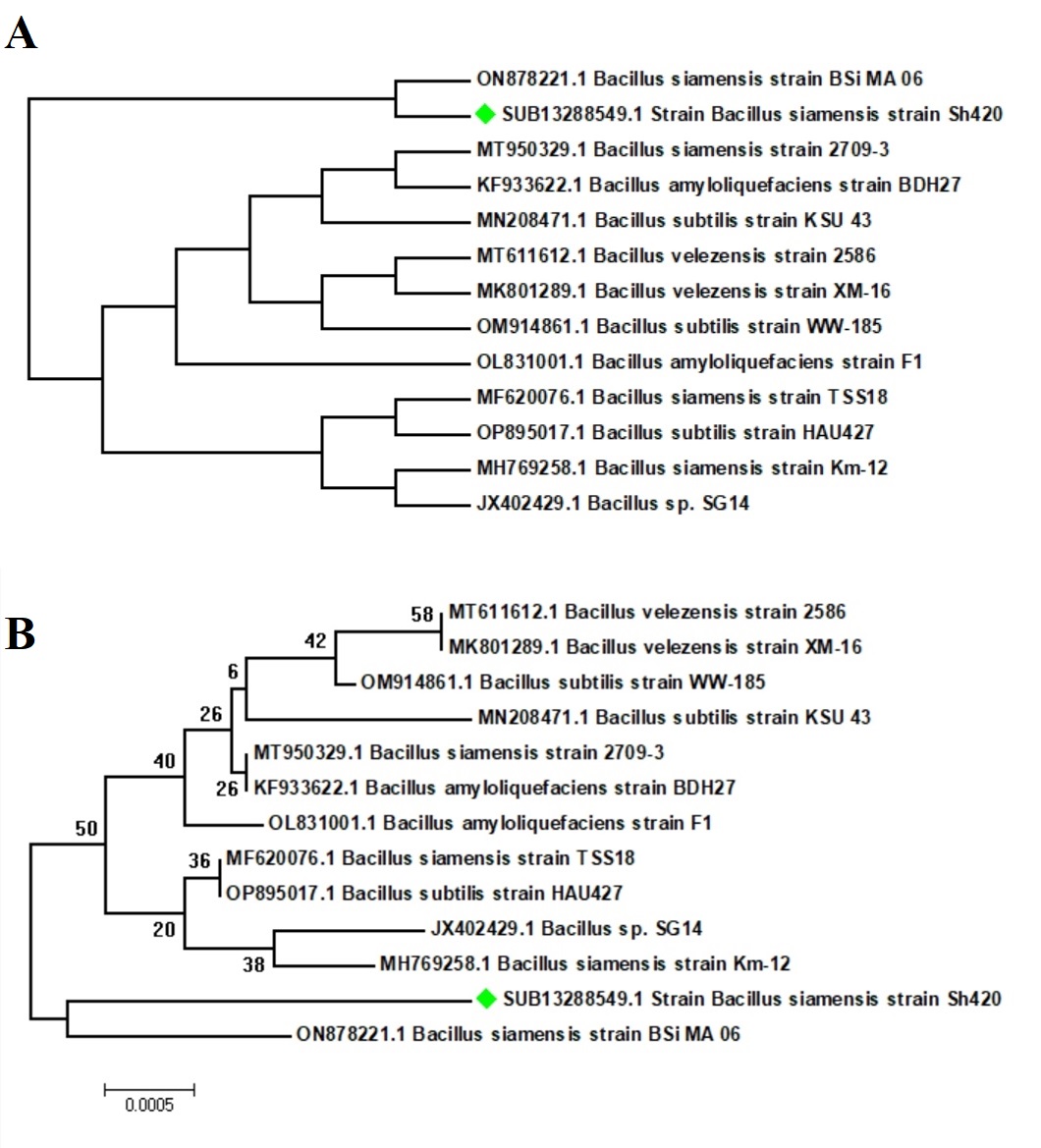


**Supplementary Figure S1:** Maximum likelihood consensus phylogenetic tree of the tested bacteria from highly conserved sequences obtained from blast for the strains Sh420. The numbers on the nodes are bootstrap values inferred from 1,000 replicates. (A) bootstrap consensus tree (B) Normal generated tree without bootstrap values.


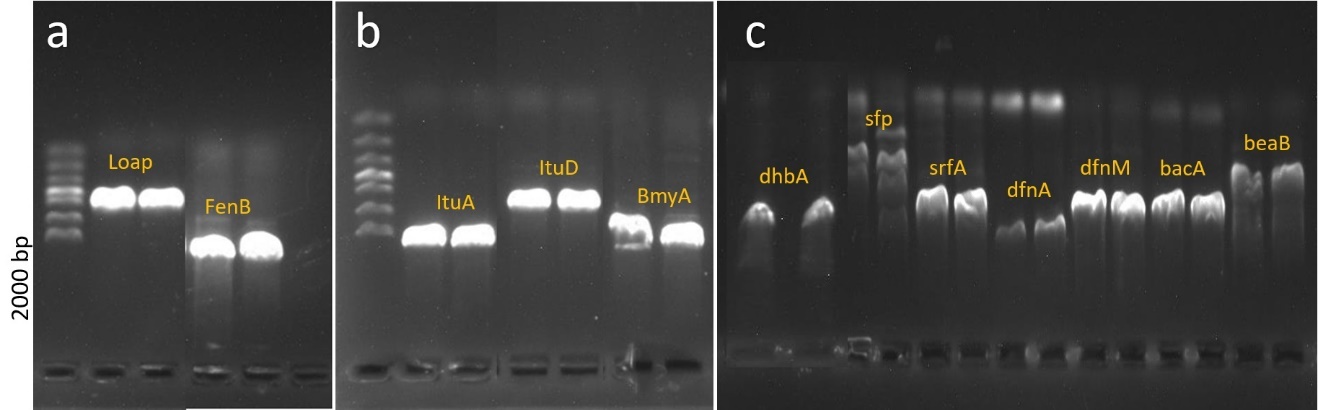


Supplementary Figure S2: PCR amplification of antifungal LPs biosynthetic genes in B. *siamensis* Sh420. Loap, regulator of antibiotic gene clusters); FenB, Fengycin; ituA, iturin; itu D, Iturin D; BmyA, Bacillomycin A; dhbA, Bacillibactin; sfp, surfactin; srfA, surfactin; dfnA, difficidin; dfnM, difficidin; bacA, Bacilysin and beaB, Bacillaene.
